# The change in pediatric subject symptoms during the COVID-19 pandemic in China: an increase in cardiac consultation

**DOI:** 10.1186/s13052-022-01384-6

**Published:** 2022-12-12

**Authors:** Yaqi Tang, Shujing Ma, Gang Luo, Zhixian Ji, Shuiyan Zhao, Yue Cao, Silin Pan

**Affiliations:** Heart Center, Qingdao Women and Children’s Hospital, Qingdao University, 6 Tongfu Road, Shibei District, Qingdao, 266034 Shandong China

**Keywords:** COVID-19, Chest pain, Palpitation, Pediatrics, Etiology, Incidence

## Abstract

**Background:**

It is reported that the adverse impact of nonpharmaceutical interventions (NPIs) on the mental health of children and adolescents may lead to psychologically related disorders during the coronavirus disease 2019 (COVID-19) period. Subject symptoms such as chest pain, chest tightness, and palpitation may be related to increased stress and anxiety in children and adolescents. The present research aimed to determine the number of pediatric consults and etiology of subject symptoms during the COVID-19 pandemic period and compared it with the same timelines in 2019 and 2021 to discuss the impact of different periods on the organic disease onset of children with subject symptoms, especially in cardiac involvement.

**Methods:**

Children who visited Qingdao Women and Children’s Hospital, Qingdao University between January 23 to April 30, 2019 (pre-COVID-19 period), January 23 to April 30, 2020 (COVID-19 period), and January 23 to April 30, 2021 (post-COVID-19 period) presenting chest pain, chest tightness, and palpitation were recruited. Information to determine gender, age, medical history, department for the initial visit, clinical manifestations, time from the latest onset to the visit, and diagnosis were recorded.

**Result:**

A total of 891 patients were enrolled in the present study (514 males; median age: 7.72). One hundred twenty-three patients presented during the pre-COVID-19 period while 130 during the COVID-19 period, nevertheless, the number substantially increased during the post-COVID-19 period (*n* = 638). Cardiac etiology accounted for 1.68% (*n* = 15) of the patient population, including arrhythmias (*n* = 10, 1.12%), myocarditis (*n* = 4, 0.44%), and atrial septal defect (n = 1, 0.11%). There was no significant difference among groups in the distribution of organic etiology. The median time from the latest onset to the visit during the pre-COVID-19 period was 7 days compared to 10 days during the COVID-19 period and 3 days during the post-COVID period.

**Conclusion:**

During the post-COVID-19 period, the median time from the latest onset to the visit was significantly shorter than that in the pre-COVID-19 period or COVID-19 period. The pediatric consult of children with subject symptoms presented increased substantially during the post-COVID-19 period, while there was no significant difference in the number of patients involving the cardiac disease. Clinicians ought to be more careful to screen heart diseases to prevent missed diagnosis and misdiagnosis during special periods.

## Introduction

Since the outbreak of the coronavirus disease 2019 (COVID-19) in December 2019 [1], effective nonpharmaceutical interventions (NPIs) have been enforced to reduce the transmission in China, including the closure of public places, the mandatory wearing of the mask, and restrictions on home activity [2]. Despite NPIs being essential, it exerts adverse effects on mental health that could cause clinical manifestations related to the psychology of children and adolescents [3–6].

Chest pain, chest tightness, and palpitation are the cluster of subject symptoms that can be a strained experience for parents due to the acute onset and obvious clinical manifestations, which account for more than 5.2% of all pediatric cardiology consult [7–9]. These symptoms can be the complaints of children with congenital heart disease (CHD) or peri−/myocarditis, but also occur in children with non-organic etiology [10]. The association between children and adolescents presenting in subject symptoms with increasing levels of stress and anxiety has been reported already [11–13].

During the COVID-19 pandemic in 2020, strict NPIs had been implemented in Qingdao till 2021 when NPIs were gradually relaxed. Schools and businesses have reopened successively, and people with negative results of reverse transcription-polymerase chain reaction (RT-PCR) assays for COVID-19 in 48 h have been allowed to attend limited public gatherings. However, measures including body distance and wearing masks in public places are still mandatory. Therefore, we sought to determine the number of pediatric consultations and etiology of subject symptoms during the COVID-19 pandemic period and compared it with the same timelines in 2019 and 2021.

## Methods

### Patient population

The study was conducted at Qingdao Women and Children’s Hospital, Qingdao University, China. The hospital is a provincial regional children’s medical center with a representative of the regional population. Patients with complaints of chest pain, chest tightness, and palpitation were included from January 23 to April 30, 2019, January 23 to April 30, 2020, and January 23 to April 30, 2021. All patients were evaluated by pediatric cardiologists. Information to determine sex, age, medical history, family history, history of drug allergy, clinic manifestations, associated symptoms, and diagnosis were recorded. Those who have suffered from COVID-19 or taken drugs related to COVID-19 were excluded. RT-PCR assays of COVID-19 was tested for patients who visited the hospital in 2020 and 2021.

### Statistical analysis

Continuous variables (ages, time from the latest onset to the visit) were presented as median with the interquartile range (IQR). Categorical variables (sex, department for initial visit, clinic manifestations, diagnosis) were expressed as a percentage. Patients were divided into three groups: the pre-COVID-19 period (January 23 to April 30, 2019), the COVID-19 period (January 23 to April 30, 2020), and the post-COVID-19 period (January 23 to April 30, 2021). The difference in categorical variables among groups was compared by the Pearson chi-square test, and the difference in continuous variables was compared by the rank-sum test. All data were analyzed by SPSS 25.0, and *P* value less than 0.05 was considered significant.

## Results

### Demographics and characteristics

**Fig. 1:**
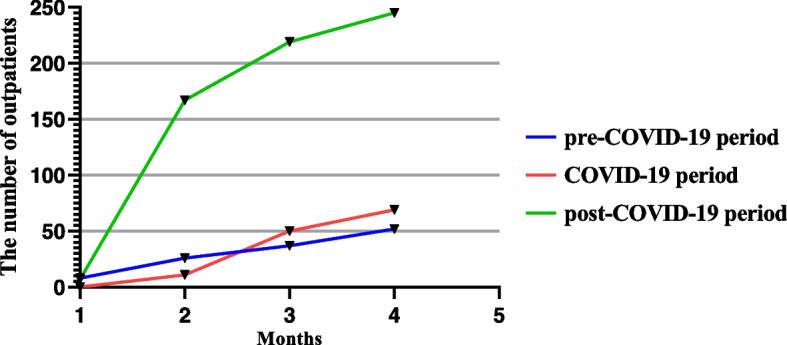
Number of children visits monthly

Eight hundred ninety-one medical records of outpatients were collected (514 males; median age: 7.72). Of these, 123 (68 males; median age: 7.31) were allocated to the pre-COVID-19 period while 130 (69 males; median age: 7.71) were allocated to the COVID-19 period, and 638 (377 males; median age: 7.85) were allocated to the post-COVID-19 period according to the visit period. There was no significant difference among groups in the distribution of median age or sex. The median time from the latest onset to the visit of patients in the COVID-19 period was 10 days compared with 7 days in the pre-COVID-19 period, while a shorter median time was found in the post-COVID-19 period (3 days). During the pre-COVID period, 35.77% (*n* = 44) of patients complained about chest pain, while the proportion rose to 44.20% (*n* = 282) in the post-COVID-19 period. There were significant differences in the distribution of symptoms among the groups. Similarly, there was no significantly different among groups in number of children with concomitant symptoms in respiratory (20.32%; 19.51%; 18.50%), gastrointestinal (7.31%; 5.38%; 5.60%), syncope (3.25%; 6.92%; 5.33%) and rash (0.81%; 0.77%; 0.47%), except those accompanying with fever (13.82%; 0.00%; 0.00%). There was no significant difference among groups in the number of children with a history of surgery, chronic disease, positive family history, and drug allergy. (Table 1). Trends in the number of patients monthly were shown in Fig. 1.

**Table 1:** Demographics and clinic manifestation of the research population. *N* = 891

Characteristic	Pre-COVID-19	COVID-19	Post-COVID-19	***P*** value
Age, years	7.31 (0.40–15.70)	7.71 (0.020–14.89)	7.85 (0.278–17.32)	0.72
Men, *n* (%)	68 (55.29)	69 (53.08)	377 (59.01)	0.37
Median visit time^a^, days	7 (3–30)	10 (3–30)	3 (1–15)	< 0.01
Cardiac symptoms, n (%)				
Chest pain	44 (35.77)	42 (32.30)	280 (43.89)	0.03
Chest tightness	61 (49.60)	75 (57.69)	313 (49.06)	
Palpitation	14 (11.38)	11 (8.46)	39 (6.11)	
Comorbidity				
Fever	17 (13.82)	0 (0.00)	0 (0.00)	< 0.01
Respiratory symptoms	25 (20.32)	24 (19.51)	118 (18.50)	0.88
Gastrointestinal symptoms	9 (7.31)	7 (5.38)	37 (5.60)	0.76
Syncope and dizzy	4 (3.25)	9 (6.92)	34 (5.33)	0.42
Rash	1 (0.81)	1 (0.77)	3 (0.47)	0.85
Medical history				
Surgery	2 (1.63)	3 (2.30)	10 (1.57)	0.84
Chronic disease	6 (4.88)	8 (6.15)	47 (7.37)	0.57
Positive family history	8 (6.50)	7 (5.38)	36 (5.64)	0.92
Drug allergy	3 (2.43)	1 (0.77)	13 (2.04)	0.57

^a^ Median visit time: the time from the latest onset to the visit

### Etiology

**Fig. 2:**
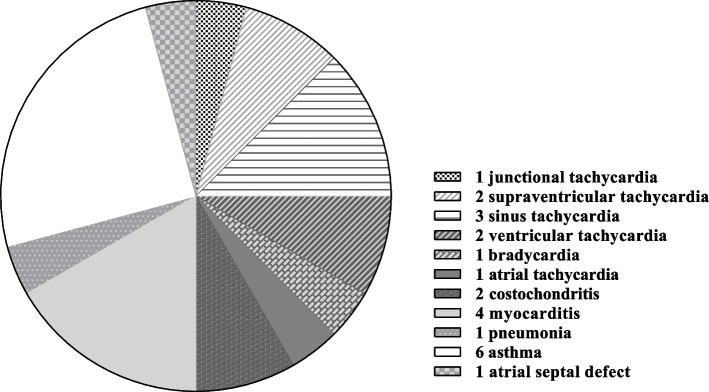
The organic etiology of patients. *N* = 24

Organic etiology including cardiac, respiratory, and musculoskeletal accounted for 2.69% (*n* = 24) of the 891 patients. Cardiac etiology accounted for 1.68% (*n* = 15) of the patient population, including arrhythmias (*n* = 10, 1.12%), myocarditis (*n* = 4, 0.44%), and atrial septal defect (n = 1, 0.11%) (Fig. 2). The proportion of children with organic etiology in the groups was 4.06% (*n* = 5), 1.54% (*n* = 2), and 2.66% (*n* = 17), respectively. There was no significant difference among groups in the distribution of organic etiology (Table 2).

**Table 2:** The frequency of children with organic etiology

Etiology, ***n*** (%)	Pre-COVID-19	COVID-19	Post-COVID-19	***P*** value
Non-organic	118 (95.93)	128 (98.46)	621 (97.34)	0.46
Organic	5 (4.06)	2 (1.54)	17 (2.66)	

### Laboratory examination

Patients who visited during 2020 and 2021 included in the present study had negative results of RT-PCR assays for COVID-19.

## Discussions

People in China have cooperated well with public preventive measures, however, such measures may also exert an adverse effect on human health. Jin et al. reported that the number of patients with allergic rhinitis and acute pharyngitis in the department of otolaryngology increased significantly from February to April 2020 compared to the previous 3 years [14]. Other studies with different inclusion times also showed an increase in pediatric consults [15, 16]. From our research, no significant increase was observed in the number of patients during the COVID-19 period compared with the pre-COVID-19 period, but it is important to note that the number increased substantially during the post-COVID-19 period. We speculated that the result may be related to the higher psychological distress and the medical care avoidance behavior during the COVID-19 period [17, 18]. Besides, researchers in several regions have pointed out the distinct decrease in the incidence of the acute coronary syndrome and pulmonary infection, while the incidence of stress cardiomyopathy increased affected by the epidemic [4, 19, 20]. In addition, the possible effect of seasonal variation in the attack of subject symptoms was explained by comparison with the control group in the corresponding period in 2019 and 2020. Besides, we found that the patient consults increased monthly in each group, nevertheless, we only focused on the impact of symptoms onset during the COVID-19 period and did not make further research on this trend.

After evaluation by the cardiologist and other pediatricians, 97.31% of the children with subject symptoms were considered non-organic etiology attacks. Similar to other studies, our results showed that only 1.68% of the patient population were diagnosed with cardiac etiology, which confirmed that most symptoms are not likely to reflect an underlying cardiac disease [21, 22]. Moreover, we found that there was no significant difference among the groups in the distribution of cardiac etiology in total clients, which indicates that the increase in subject symptoms of children may not possibly directly influence the etiology. Furthermore, there was no significant difference in concomitant symptoms and medical history of children among the three periods except for the children with fever. Due to the children who visited the hospital was required to maintain normal body temperature during the post−/COVID-19 period, we were unable to make a reasonable comparison in children with fever. However, excluding the impact of season and the population of children, it could be determined that the number of children with symptoms of suspected cardiac involvement increased in the post-COVID-19 period with no increase or decrease in the etiology related to cardiac disease. This trend has undoubtedly increased the workload of clinicians. Therefore, clinicians ought to be more careful to screen heart diseases to prevent missed diagnosis and misdiagnosis. Auxiliary examination such as echocardiography (Echo) needs to be performed targeted rather than universal. American College of Cardiology (ACC) has established an Appropriate Use Criteria (AUC) for Echo in patients with chest pain or palpitations [23], and be proven effective by other studies [24–26]. More research is demanded to be carried out to determine the diagnostic process of these subjects.

Compared with children in the other groups, our study showed that the median time from the latest onset to the visit was prolonged during the COVID-19 period. The delay has also been found in other studies. Yoshimoto [27] et al. pointed out that delay in reperfusion therapy increased from March 2019 to February 2020 compared with March 2020 to February 2021 in patients with acute ischemic stroke. Fardman [28] et al. reported that compared with March 9 to April 30, 2018, the total ischemic time of patients with ST-elevated myocardial infarction admitted to the hospital during the parallel phase in 2020 was prolonged. Interestingly, we found that during the post-COVID-19 period, the median time from the latest onset to the visit was significantly shorter than that in the pre-COVID-19 period or COVID-19 period. This disparity may be related to the attention paid to children by their parents during the post-COVID-19 period [29]. In special periods, parents may be more careful or nervous about symptoms of suspected cardiac involvement in children. We encourage attention due to the symptoms that may be related to cardiac diseases and COVID-19, however, clinicians ought to comfort children and parents properly after confirming the exclusion of organic diseases to relieve the stress and anxiety [30].

Considering that there was no psychological evaluation, we did not discuss the association between psychological stress and presenting subject symptoms in children. Therefore, we have not made a further study for the increased number of children during the post-COVID-19 period, which is the deficiency of the research.

## Conclusions

The present study reported that increase in pediatric consults of children with subject symptoms presented during the post-COVID-19 period with a less median time from the latest onset to the visit to the pediatric center. Similar to previous studies, the majority of subject symptoms in children were shown in favor of non-organic etiology. Despite the substantial increase in the number of patients during the post-COVID-19 period, there was no significant difference in patients involving cardiac disease. Clinicians ought to be more careful to screen heart diseases to prevent missed diagnosis and misdiagnosis when the number of children with subject symptoms increases during the post-COVID-19 period.

## Data Availability

The datasets used and/or analyzed during the current study are available from the corresponding author upon reasonable request.

## Abbreviations

COVID-19: Coronavirus disease 2019
NPIs: Nonpharmaceutical interventions
RT-PCR: Reverse transcription-polymerase chain reaction
IQR: Interquartile range

## References

[1] Zhu N, Zhang D, Wang W (2020). A novel coronavirus from patients with pneumonia in China, 2019. N Engl J Med.

[2] Liu P, Xu M, Lu L (2022). The changing pattern of common respiratory and enteric viruses among outpatient children in Shanghai, China: two years of the COVID-19 pandemic. J Med Virol..

[3] Sacco E, Gandi C, Li Marzi V (2021). Extensive impact of COVID-19 pandemic on pelvic floor dysfunctions care: a nationwide interdisciplinary survey. Neurourol Urodyn.

[4] Jabri A, Kalra A, Kumar A (2020). Incidence of stress cardiomyopathy during the coronavirus disease 2019 pandemic. JAMA..

[5] Goldberg L, Ziv A, Vardi Y (2022). The effect of COVID-19 pandemic on hospitalizations and disease characteristics of adolescents with anorexia nervosa. Eur J Pediatr.

[6] Saunders NR, Kurdyak P, Stukel TA (2022). Utilization of physician-based mental health care services among children and adolescents before and during the COVID-19 pandemic in Ontario, Canada. JAMA Pediatr.

[7] Geggel RL (2004). Conditions leading to pediatric cardiology consultation in a tertiary academic hospital. Pediatrics..

[8] Kroenke LK, Arrington ME, Mangelsdorff AD (1990). The prevalence of symptoms in medical outpatients and the adequacy of therapy. Arch Intern Med.

[9] Petrie KJ, Faasse K, Crichton F, Grey A (2014). How common are symptoms? Evidence from a New Zealand national telephone survey. BMJ Open.

[10] Blücher S, Kaemmerer H, Lammers A, Brodherr-Heberlein S, Hess J (2000). Event recorder for etiological evaluation of sporadically occurring cardiovascular complaints and symptoms. Herz..

[11] Lin CH, Lin WC, Ho YJ, Chang JS (2008). Children with chest pain visiting the emergency department. Pediatr Neonatol.

[12] Ginsburg GS, Riddle MA, Davies M (2006). Somatic symptoms in children and adolescents with anxiety disorders. J Am Acad Child Adolesc Psychiatry.

[13] Kenar A, Örün UA, Yoldaş T (2019). Anxiety, depression, and behavioural rating scales in children with non-cardiac chest pain. Cardiol Young.

[14] Jin L, Fan K, Tan S (2021). Analysis of the characteristics of outpatient and emergency diseases in the department of otolaryngology during the “COVID-19” pandemic. Sci Prog.

[15] Kang HM, Jeong DC, Suh BK, Ahn MB (2021). The impact of the coronavirus Disease-2019 pandemic on childhood obesity and vitamin D status. J Korean Med Sci.

[16] Kalem M, Özbek EA, Kocaoğlu H (2021). The increase in paediatric orthopaedic trauma injuries following the end of the curfew during the COVID-19 period. J Child Orthop.

[17] Mzadi AE, Zouini B, Kerekes N, Senhaji M (2022). Mental health profiles in a sample of Moroccan high school students: comparison before and during the COVID-19 pandemic. Front Psychiatry.

[18] Papafaklis MI, Katsouras CS, Tsigkas G (2020). “Missing” acute coronary syndrome hospitalizations during the COVID-19 era in Greece: medical care avoidance combined with a true reduction in incidence?. Clin Cardiol.

[19] Barschkett M, Koletzko B, Spiess CK (2021). COVID-19 associated contact restrictions in Germany: marked decline in Children’s outpatient visits for infectious diseases without increasing visits for mental health disorders. Child Basel Switz.

[20] Perrin N, Iglesias JF, Rey F, et al. Impact of the COVID-19 pandemic on acute coronary syndromes. Swiss Med Wkly. 2020;150:w20448. 10.4414/smw.2020.20448. 10.4414/smw.2020.20448 33382905

[21] Sumski CA, Goot BH (2020). Evaluating chest pain and heart murmurs in Pediatric and adolescent patients. Pediatr Clin N Am.

[22] Sedaghat-Yazdi F, Koenig PR (2014). The teenager with palpitations. Pediatr Clin N Am.

[23] Campbell RM, Douglas PS, Eidem BW (2014). ACC/AAP/AHA/ASE/HRS/SCAI/SCCT/SCMR/SOPE 2014 appropriate use criteria for initial transthoracic echocardiography in outpatient Pediatric cardiology. J Am Coll Cardiol.

[24] Sheth S, Fares M, Kikano S (2021). Appropriate use of echocardiography for palpitations in paediatric cardiology clinics. Cardiol Young.

[25] Chamberlain RC, Pelletier JH, Blanchard S (2017). Evaluating appropriate use of Pediatric echocardiograms for chest pain in outpatient clinics. J Am Soc Echocardiogr.

[26] Dasgupta S, Kelleman M, Sachdeva R (2020). Application of appropriate use criteria for echocardiography in Pediatric patients with palpitations and arrhythmias. Pediatr Qual Saf.

[27] Yoshimoto T, Shiozawa M, Koge J (2021). Evaluation of workflow delays in stroke reperfusion therapy: a comparison between the year-long pre-COVID-19 period and the with-COVID-19 period. J Atheroscler Thromb..

[28] Fardman A, Zahger D, Orvin K (2021). Acute myocardial infarction in the Covid-19 era: incidence, clinical characteristics and in-hospital outcomes-a multicenter registry. PLoS One.

[29] Russell BS, Hutchison M, Tambling R, Tomkunas AJ, Horton AL (2020). Initial challenges of caregiving during COVID-19: caregiver burden, mental health, and the parent-child relationship. Child Psychiatry Hum Dev.

[30] Li Y, Deng H, Wang H (2022). Building the mental health management system for children post COVID-19 pandemic: an urgent focus in China. Eur Child Adolesc Psychiatry..

